# Bilingualism Is Associated with a Delayed Onset of Dementia but Not with a Lower Risk of Developing it: a Systematic Review with Meta-Analyses

**DOI:** 10.1007/s11065-020-09426-8

**Published:** 2020-02-08

**Authors:** Stefano Brini, Hamid R. Sohrabi, Jeffrey J. Hebert, Mitchell R. L. Forrest, Matti Laine, Heikki Hämäläinen, Mira Karrasch, Jeremiah J. Peiffer, Ralph N. Martins, Timothy J. Fairchild

**Affiliations:** 1 Discipline of Psychology, Exercise Science, Chiropractic and Counselling, Murdoch University, Perth, Western Australia Australia; 2 Turku Brain and Mind Center, Turku, Finland; 3 Health Services Research and Management School of Health Sciences, City, University of London, London, UK; 4 School of Medical and Health Sciences, Edith Cowan University, Perth, Western Australia Australia; 5 Department of Biomedical Sciences, Macquarie University, Macquarie Park, New South Wales Australia; 6 Faculty of Kinesiology, University of New Brunswick, Fredericton, Canada; 7 Department of Psychology, Åbo Akademi University, Turku, Finland; 8 Australian Alzheimer’s Research Foundation, Perth, Western Australia Australia; 9 Centre for Molecular Medicine and Innovative Therapeutics, Murdoch University, Perth, Western Australia Australia; 10 Department of Psychology and Speech-Language Pathology, University of Turku, Turku, Finland

**Keywords:** Bilingualism, Multilingualism, Mild cognitive impairment, Dementia, Alzheimer’s disease, Meta-analysis

## Abstract

Some studies have linked bilingualism with a later onset of dementia, Alzheimer’s disease (AD), and mild cognitive impairment (MCI). Not all studies have observed such relationships, however. Differences in study outcomes may be due to methodological limitations and the presence of confounding factors within studies such as immigration status and level of education. We conducted the first systematic review with meta-analysis combining cross-sectional studies to explore if bilingualism might delay symptom onset and diagnosis of dementia, AD, and MCI. Primary outcomes included the age of symptom onset, the age at diagnosis of MCI or dementia, and the risk of developing MCI or dementia. A secondary outcome included the degree of disease severity at dementia diagnosis. There was no difference in the age of MCI diagnosis between monolinguals and bilinguals [mean difference: 3.2; 95% confidence intervals (CI): −3.4, 9.7]. Bilinguals vs. monolinguals reported experiencing AD symptoms 4.7 years (95% CI: 3.3, 6.1) later. Bilinguals vs. monolinguals were diagnosed with dementia 3.3 years (95% CI: 1.7, 4.9) later. Here, 95% prediction intervals showed a large dispersion of effect sizes (−1.9 to 8.5). We investigated this dispersion with a subgroup meta-analysis comparing studies that had recruited participants with dementia to studies that had recruited participants with AD on the age of dementia and AD diagnosis between mono- and bilinguals. Results showed that bilinguals vs. monolinguals were 1.9 years (95% CI: −0.9, 4.7) and 4.2 (95% CI: 2.0, 6.4) older than monolinguals at the time of dementia and AD diagnosis, respectively. The mean difference between the two subgroups was not significant. There was no significant risk reduction (odds ratio: 0.89; 95% CI: 0.68–1.16) in developing dementia among bilinguals vs. monolinguals. Also, there was no significant difference (Hedges’ *g* = 0.05; 95% CI: −0.13, 0.24) in disease severity at dementia diagnosis between bilinguals and monolinguals, despite bilinguals being significantly older. The majority of studies had adjusted for level of education suggesting that education might not have played a role in the observed delay in dementia among bilinguals vs. monolinguals. Although findings indicated that bilingualism was on average related to a delayed onset of dementia, the magnitude of this relationship varied across different settings. This variation may be due to unexplained heterogeneity and different sources of bias in the included studies. Registration: PROSPERO CRD42015019100.

## Key Points

- Meta-analytic results showed that bilinguals vs. monolinguals were older at the time of Alzheimer’s symptom onset and dementia diagnosis.
- Meta-analytic results did not show a reduction in the risk of developing dementia among bilinguals vs. monolinguals.
- Several sources of bias were identified, including poor measurement of participants’ language profiles with analytic samples including differing dementia aetiologies.

## Introduction

### Rationale

Approximately 43.8 million people lived with dementia worldwide in the year 2016 (Nichols et al., 2019) and this number is projected to increase to 115.5 million people by 2050 (Prince et al., 2013). The global economic cost of dementia is estimated to surpass US$2 trillion per year by 2030 (Wimo et al., 2017). A five-year delay in the onset of Alzheimer’s disease (AD), the most common form of dementia, could reduce the number of patients living with the disease worldwide by 57%, thereby alleviating the associated economic costs by half (Sperling et al., 2011). Therefore, identifying modifiable lifestyle factors that can slow or delay the onset of dementia is a world’s public health priority (WHO, 2017; Wortmann, 2012).

One such factor may be bilingualism, which is the ability to speak two languages (Luk & Bialystok, 2013). This hypothesis comes from studies showing that bilinguals develop mild cognitive impairment (MCI), dementia, and AD, 4–7 years later than monolinguals (Alladi et al., 2013; Bialystok, Craik, Binns, Ossher, & Freedman, 2014; Bialystok, Craik, & Freedman, 2007). Others, however, have not documented such differences (Lawton, Gasquoine, & Weimer, 2015; Yeung, John, Menec, & Tyas, 2014). Also, while longitudinal prospective studies showed no risk reduction among bilinguals relative to monolinguals (Ljungberg, Hansson, Adolfsson, & Nilsson, 2016; Yeung et al., 2014; Zahodne, Schofield, Farrell, Stern, & Manly, 2014), foreign language education during adolescence has been associated with reduced risk of MCI later in life (Wilson, Boyle, Yang, James, & Bennett, 2015). Some authors have argued that confounding factors including migration status and education may explain some differences in study outcomes in cross-sectional and longitudinal studies (Fuller-Thomson, 2015; Fuller-Thomson & Kuh, 2014).

One systematic review concluded that “public health policy should… remove recommendations regarding bilingualism as a strategy to delay dementia” (Mukadam, Sommerlad, & Livingston, 2017). However, the authors conducted a meta-analysis of only four longitudinal prospective studies without performing meta-analyses on cross-sectional reports. Moreover, while studies without a monolingual control group were excluded from this review (Mukadam et al., 2017), their meta-analysis included one study (Sanders, Hall, Katz, & Lipton, 2012) which did not clearly define the control group as monolingual. That review did not include age at MCI diagnosis as an outcome or studies published more recently (Hack, Dubin, Fernandes, Costa, & Tyas, 2019; Ljungberg et al., 2016; Perani et al., 2017; Ramakrishnan et al., 2017; Zheng et al., 2018). As such, before suggesting that bilingualism should not be recommended as a strategy for delaying dementia, a careful re-evaluation of the available evidence is necessary (Del Maschio, Fedeli, & Abutalebi, 2018).

### Objectives

Differences in study outcomes in the field of bilingualism and dementia research as well as the need to identify strategies to delay the onset of dementia as highlighted in the *Global plan on the public health response to dementia 2017–2025* by the World Health Organization (WHO, 2017) prompted this systematic review. We assessed whether bilingualism relative to monolingualism might delay the age at which participants experienced the initial symptoms of AD and delay the age at which participants were diagnosed with MCI or dementia. We also examined whether bilingualism might be associated with a lower risk of dementia. The primary objectives were to review cross-sectional and longitudinal prospective studies investigating (i) differences in the age of symptom onset and age at diagnosis of MCI or dementia between older monolinguals and bilinguals, and (ii) the relationship between bilingualism relative to monolingualism and risk of dementia in older cognitively intact adults. A secondary objective was to investigate differences in disease severity at dementia diagnosis between older monolinguals and bilinguals.

## Methods

### Search Strategy and Selection Criteria

This systematic review with meta-analyses accords with the Preferred Reporting Items for Systematic Reviews and Meta-Analyses Statement (PRISMA; Moher, Liberati, Tetzlaff, & Altman, 2010). Eligible studies had to compare monolingual to bilingual participants on at least one of the following outcomes: reported age of symptom onset or age at diagnosis for MCI or dementia, degree of cognitive impairment at dementia diagnosis, or risk of dementia or MCI. Given the lack of a clear uniform definition of bilingualism in the literature, we included studies independently of the way bilingualism was operationalized or measured, or whether proficiency in the second language had been objectively assessed. We included studies that had recruited participants with MCI or dementia as assessed using clinical measures such as the National Institute of Neurological and Communicative Disorders and Stroke and the Alzheimer’s Disease and Related Disorders Association (NINCDS-ADRDA) as well as cognitively intact individuals. We excluded studies without a group of monolinguals. We also excluded reports, conference abstracts, reviews, commentaries, editorials, letters, news articles, case series, and discussion forums, as well as grey literature including non-peer reviewed empirical studies. We searched cross-sectional, prospective, case-control studies, and randomized controlled trials across several databases including CINHAL, The Cochrane Library, PubMed, PsycINFO, LILACS, and Embase. Filters were used to exclude animal studies, but no restrictions were placed on time and language. The initial search was performed on September 3, 2015 and refreshed several times with the last refresh being complete on December 5, 2018. We used similar keywords and criteria for each search.

The database searches were conducted by S.B., while the screening for title and abstract as well as the full-text screening was conducted independently by pairs of review authors (S.B., T.J.F., and J.J.H.). Data extraction was completed independently by pairs of review authors (S.B., M.F., T.J.F., and J.P.). We used Covidence software for each of these steps (Innovation, 2017). Disagreements were resolved through consensus and discussion with a third review author. We requested additional information from corresponding authors when necessary. Details of the protocol for this systematic review were registered a priori (PROSPERO 2015 CRD42015019100).

#### Embase search strategy (example)

‘dementia’/exp. OR ‘dementia’ OR ‘Alzheimer disease’/exp. OR ‘Alzheimer disease’ OR ‘frontotemporal dementia’/exp. OR ‘frontotemporal dementia’ OR ‘multiinfarct dementia’/exp. OR ‘multiinfarct dementia’ OR ‘mild cognitive impairment’/exp. OR ‘mild cognitive impairment’ OR ‘memory disorder’/exp. OR ‘memory disorder’ OR ‘Parkinson disease’/exp. OR ‘parkinson disease’ AND (‘multilingualism’/exp. OR ‘multilingualism’) OR ‘multilingualism’/exp. OR ‘multilingualism’ OR ‘bilingualism’/exp. OR ‘bilingualism’ OR ‘English as a second language’/exp. OR ‘English as a second language’ AND [article]/lim AND ([adult]/lim OR [middle aged]/lim OR [aged]/lim OR [very elderly]/lim) AND [humans]/lim.

### Study Selection

**Fig. 1:**
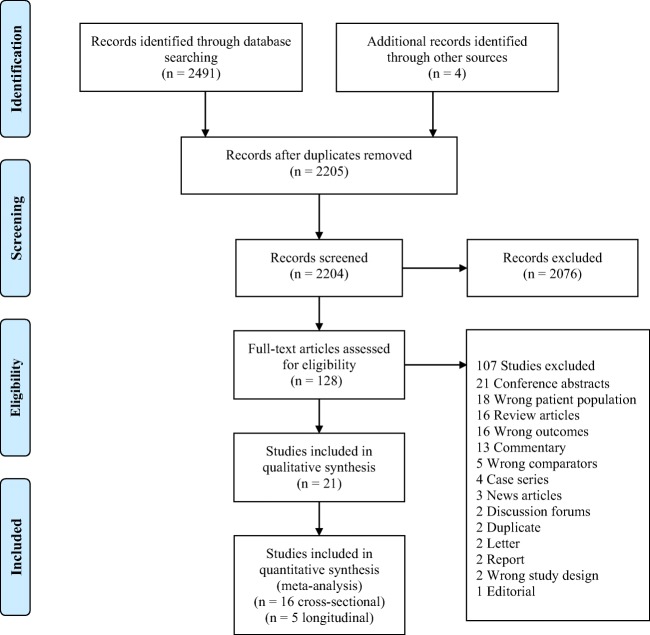
Prisma flow diagram showing the final number of included studies meeting selection criteria according to PRISMA (Preferred Reporting Items for Systematic reviews and Meta-Analyses) 2009

The number of studies screened and included for quantitative synthesis is presented in Fig. 1. The quantitative synthesis included three cross-sectional studies with age at MCI diagnosis (Table 1) and 16 cross-sectional studies with age at AD symptom onset and age at dementia or AD clinical diagnosis (Table 2). There was one longitudinal prospective study with the risk of MCI as the outcome (Table 3) and five with the risk of dementia as the outcome (Table 4).

**Table 1:** Cross-sectional studies investigating the relationship between bilingualism and MCI.

	**Study characteristics**						**Effect sizes (MD = BL age minus ML age, 95%: CI)**		
**Study**	**N (% of females)**	**Mean age of diagnosis & symptom onset**	**Education level**	**Language measure**	**MCI diagnosis and type**	**MMSE scores**	**Age of diagnosis**	**Age of onset**	**Cognitive impairment**
Bialystok et al., 2014	ML: 28 (50%); BL: 26 (56%)	ML: 66.5 (12.3) BL: 70.0 (10.7)	ML: 15.5 (3.8) BL: 14.3	LSBQ	MCI	ML: 29 (1.4) BL: 28.4 (1.9)	3.5 (−1.77–8.77)	4.7 (0.97–10.37)	0.6 (−0.17–1.37)
Ossher et al. 2012	ML (SDaMCI): 49 (55%) BL (SDaMCI): 19 (32%) ML (MDaMCI): 22 (45%) BL (MDaMCI): 21 (43%)	ML (SDaMCI): 74.9 (6.9) BL (SDaMCI): 79.4 (6.3) ML (MDaMCI): 75.2 (8.5) BL (MDaMCI): 72.6 (7.2)	ML (SDaMCI): 14.7 (2.5) BL (SDaMCI): 14.5 (3.9) ML (MDaMCI): 14.9 (3.3) BL (MDaMCI): 15.0 (3.3)	Questionnaire	Clinical interview including neuropsychological tests; SDaMCI, MDaMCI	ML (SDaMCI): 27.7 (1.6) BL (SDaMCI): 27.6 (1.9) ML (MDaMCI): 27.9 (1.4) BL (MDaMCI): 27.7 (1.8)	SDaMCI: 4.50 (0.93–8.07) MDaMCI: −2.60 (−7.32–2.12)	NA	SDaMCI: −0.1 MDaMCI: −0.2
Ramakrishnan et al., 2017	ML: 22 (18.2%) BL: 93 (20.4%)	ML: 58.1 (11.4); 55.8 (12.2) BL: 65.2 (9.9); 63.2 (10.1)	ML: 10.4 (3.7) BL: 15.5 (3.3)	NA	Clinicians used Petersen criteria for final diagnosis; MCI: amnestic MCI & non-amnestic MCI	NA	7.1 (2.36–11.84)	7.4 (2.46–12.34)	NA

ML: Monolinguals; BL: Bilinguals; Mild Cognitive Impairment; SDaMCI: Single domain amnestic MCI; MDaMCI: Multiple domain MCI; LSBQ Language and Social Background Questionnaire; MMSE: Mini-Mental State Examination; CI: Confidence Intervals; MD: Mean Difference; NA: Not Available

**Table 2:** Cross-sectional studies on the relationship between bilingualism and dementia or AD.

	**Study characteristics**						**Effect sizes (MD = BL age minus ML age, 95%: CI)**		
**Study**	**N (% of females)**	**Mean age of diagnosis & symptom onset**	**Education level**	**Language measure**	**Dementia diagnosis and type**	**MMSE/3MSE scores**	**Age of diagnosis**	**Age of onset**	**Cognitive impairment**
Alladi et al., 2017	ML: 72 (52.8%) BL: 121 (36.4%)	Dementia ML: 61.0 (9.5), 58.4 (9.3) BL: 64.2 (9.4), 61.7 (9.1)	ML: 6.9 (5.3) BL: 13.9 (4.3)	Case records	MMSE, ACE-R, FrSBe; bvFTD, PNFA, SD, FTD-MND, CBD, PSP	ML: 15.9 (10.3) BL: 18.1 (10.2)	FTD 3.2 (0.43–5.97)	FTD 3.3 (0.61–5.99)	FTD 2.2 (−0.81–5.21)
Alladi et al., 2013	ML: 257 (49.0%) BL: 391(25.1%)	Dementia ML: 63.4 (11.4), 61.1 (11.4) BL: 68.1 (10.0), 65.6 (10.0) AD (onset) ML: 65.4 (10.0) BL: 68.6 (9.6)	ML: 5.9 (5.1) BL: 12.9 (4.9)	Family member interview	DSM-IV; AD, VaD, mixed AD with CVD, FTD, DLB	ML: 16.7 (7.5) BL: 18.9 (8.0)	Dementia 4.7 (3.03–6.37) AD 3.2 (0.67–5.73)	Dementia 4.5 (2.83–6.17) AD NA	Dementia NA AD 2.2 (0.97–3.43)
Bialystok et al., 2014	ML: 35 (54%) BL: 40 (55%)	ML: 74.2 (11.2), 70.9 (11.0) BL: 81.4 (8.4), 78.2 (8.9)	ML: 12.5 (3.7) BL: 12.2 (4.9)	LSBQ	NA; Probable AD	ML: 23.4 (3.8) BL: 22.3 (4.5)	7.2 (2.68–11.72)	7.3 (2.72–11.88)	1.1 (−0.83–3.03)
Bialystok 2007	ML: 91 (53%) BL: 93 (59%)	Dementia ML: 75.4 (9.3); 71.4 (9.6) BL: 78.6 (8.4); 75.5 (8.5) AD ML: 75.8 (9.8) BL: 79.2 (8.7)	ML: 12.4 (3.8) BL: 10.8 (4.2)	Medical records	NINCDS–ADRDA; AD, possible AD, dementia due to other neurodegenerative disorders, and CVD	ML: 21.3 (6.4) BL: 20.1 (7.1)	Dementia 3.2 (0.62–5.78) AD 3.3 (0.241–6.505)	4.1 (1.4–6.74)	AD 1.2 (−3.24–5.64)
Chertkow et al., 2010	ML: 379 (63%) BL: 253 (51%)	ML: 76.7 (7.8) BL: 77.6 (7.2)	ML: 10.9 (3.5) BL: 10.7 (3.8)	Patient & caregiver interviews	NINCDS-ADRDA; probable AD	ML: 23.1 (3.9) BL: 22.9 (4.3)	0.9 (− 0.31–2.11)	NA	0.2 (−0.45–0.85)
Clare et al., 2016	ML: 49 (45%) BL: 37 (57%)	ML: 76.2 (8.8), 73.7 (9.9) BL: 79.3 (6.8), 76.9 (7.1)	ML: 12.31 (3.04) BL: 11.84 (2.46)	LQ-SV	ICD-10; AD	ML: 23.90 (3.19) BL: 22.68 (3.16)	1.22 (−0.16–2.60)	3.21 (−0.65–7.07)	1.94 (−1.33–5.21)
Craik, Bialystok, & Freedman 2010	ML: 109 (55%) BL: 102 (59%)	ML: 76.5 (10), 72.6 (10.0) BL: 80.8 (7.7), 77.7 (7.9)	ML: 12.6 (4.1) BL: 10.6 (5.1)	NA	NINCDS-ADRDA; probable AD	ML: 21.5 (5.7) BL: 20.4 (5.6)	4.3 (1.87–6.63)	5.1 (2.64–7.56)	1.1 (−0.43–2.63)
Lawton et al., 2015	ML: 54 (65%) BL: 27 (63%)	ML: 81.10 (NA) BL: 79.31 (NA)	ML: 4.99 (4.17) BL: 7.70 (4.88)	ARSMA-II	ADDTC NINCDS–ADRDA; VaD Possible and probable AD	ML: 78.87 (9.90) BL: 79.56 (15.57)	1.79 (−4.55–0.97)	NA	0.69 (−4.97–6.35)
Perani er al., 2017	ML: 40 (52.5%) BL: 45 (71%)	ML: 71.4 (4.9) BL: 77.1 (4.5)	ML: 10.5 (4.07) BL: 8.26 (4.55)	Questionnaire	NIAAA; probable AD	ML: 21.10 (4.84) BL: 22.40 (4.19)	5.70 (3.71–7.71)	NA	1.3 (−0.65–3.25)
Schweizer et al., 2012	ML: 19 (70%) BL: 20 (70%)	ML: 77.3 (6.8) BL: 78.9 (7.7)	ML: 13.6 (3.5) BL: 11.6 (4.5)	Interview with patient and significant-other	CDR; probable AD	ML: 23.2 (3) BL: 22.1 (5.1)	1.60 (−2.95–6.15)	NA	−1.10 (−3.87–1.67)
Woumans et al., 2015	ML: 69 (69%) BL: 65 (69%)	ML: 72.5 (9.4) BL: 77.3 (10.5)	ML: 13.5 (2.8) BL: 14.7 (3.1)	Patient and caregiver interviews using Likert scale	Neurologist in consultation with a neuropsychologist; AD	ML: 24.2 (3.1) BL: 23.8 (3.4)	4.80 (1.43–8.17)	4.6 (1.17–8.03)	0.40 (−0.70–1.50)
Zheng et al., 2018	ML (Cantonese): 48 (85%) ML (Mandarin): 20 (45%) BL: 61 (57%)	Diagnosis ML (Cantonese): 67.7 (9.9) ML (Mandarin): 67.0 (9.1) BL: 74.4 (9.4) Onset ML (Cantonese): 63.9 (9.7) ML (Mandarin): 63.4 (8.9) BL: 70.9 (9.4)	ML (Cantonese): 4.92 (3.85) ML (Mandarin): 10.95 (3.28) BL: 10.79 (4.32)	BAT	Two neurologists delivered the diagnosis using the NINCDS–ADRDA; probable AD	ML (Cantonese): 12.25 (5.39) ML (Mandarin): 15.75 (6.75) BL: 16.43 (6.46)			
	**N (% of females)**	**Mean age**	**Education level**	**Language measure**	**Dementia diagnosis and type**	**MMSE/3MSE scores**	**Prevalence**		**Rate ratio**
Estanga et al., 2017	ML: 100 (58%) Early BL: 81 (54.3%) Late BL: 97 (60.8%)	ML: 57.82 (6.42) Early BL: 56.82 (6.48) Late BL: 57.56 (6.57)	ML: 12.33 (3.37) Early BL: 14.35 (3.76) Late BL: 14.98 (3.77)	BLPQ	Cognitively intact	ML: 28.44 (1.34) Early BL: 28.81 (1.09) Late BL: 28.81 (1.09)	ML: (stage 1: 11.9%; stage 2: 6.8%; and SNAP: 6.8%); Early BL: (stage 1: 3.6%; stage 2: 1.8%; and SNAP: 1.8%)		The prevalence of subjects in preclinical AD stage 1 (abnormal amyloid), stage 2 (abnormal amyloid and tau), and SNAP (abnormal tau) was significantly different (*p* = 0.02) between early bilinguals (stage 1: 3.6%; stage 2: 1.8%; and SNAP: 1.8%) and monolinguals (stage 1: 11.9%; stage 2: 6.8%; and SNAP: 6.8%)
Yeung et al., 2014	ML: 913 (60.4%) BL: 81 (61.7%) ESL: 622 (57.4%)	ML: 77.4 (6.7) BL: 77.0 (6.5) ESL: 77.1 (7.1)	ML: 10.4 (2.9) BL: 11.9 (4.2) ESL: 8.1 (3.7)	Self-repot	DSM-IIIR; Dementia	ML: 89.0 (8.1) BL: 89.3 (6.7) ESL: 83.3 (11.0)	ML: 197 (31%), 440 (69%); BL: 86 (20%), 344 (80%)		Bilingualism was not associated with risk of developing dementia

ML: Monolinguals; BL: Bilinguals; AD: Alzheimer’s Disease; FTD: Frontotemporal Dementia; bvFTD: behavioural variant Frontotemporal Dementia; PNFA: Progressive Non Fluent Aphasia; SD: Semantic Dementia; FTD-MND: Frontotemporal dementia-motor neuron disease; CBD: Cortico-Basal Degeneration; PSP: Progressive Supranuclear Palsy; VaD: Vascular Dementia; CVD: Cardiovascular Disease; DLB: Dementia with Lewy bodies; SNAP: Suspected Non-Alzheimer Pathophysiology; LSBQ Language and Social Background Questionnaire; LQ-SV Language Questionnaire – Short Version; ARSMA-II Acculturation Rating Scale for Mexican Americans; BLPQ Bilingual Language Profile Questionnaire; BAT Bilingual Aphasia Test; MMSE: Mini-Mental State Examination; 3MS: Modified Mini-Mental State; DSM-IV: Diagnostic and Statistical Manual of Mental Disorders Version Four; (ADDTC) NINCDS-ADRDA: (Alzheimer Disease Diagnostic and Treatment Centers); National Institute of Neurological and Communicative Disorders and Stroke and the Alzheimer’s Disease and Related Disorders Association; ICD-10: International Statistical Classification of Diseases and Related Health Problems; CDR: Clinical Dementia Rating; DSM-IIIR: Diagnostic and Statistical Manual of Mental Disorders, Third Edition, Revised; NA: CI: Confidence Intervals; MD: Mean Difference; Not Available

**Table 3:** Longitudinal prospective study investigating the relationship between bilingualism and MCI.

	**Study characteristics**		**Effect size**	
**Study**	**N (% female); mean age (SD), Ed, MMSE/3MS**	**Language measure, dementia diagnosis and type**	**% with dementia ML/BL % no dementia ML/BL**	**RR, OR, HR**
Wilson et al., 2015	964 (76.8%), 78.7 (7.4), 14.6 (3.2)	Self-report NINCDS–ADRDA MCI	During a mean of 5.8 years (*SD* = 3.5) of annual follow-up evaluations, 396 individuals (41.1%) developed MCI	Higher levels (>4 years) of foreign language instruction: HR = 0.687, 95% CI: 0.482, 0.961

SD: Standard Deviation; Ed: Education; MMSE: Mini-Mental State Examination; 3MS: Modified Mini-Mental State Examination; ML: Monolinguals; BL: Bilinguals; RR: Relative Risk; OR: Odds Ratios; HR: Hazard Ratios; NINCDS–ADRDA: (Alzheimer Disease Diagnostic and Treatment Centers) National Institute of Neurological and Communicative Disorders and Stroke and the Alzheimer’s Disease and Related Disorders Association; MCI: Mild Cognitive Impairment

**Table 4:** Longitudinal prospective studies investigating the relationship between bilingualism and dementia.

	**Study characteristics**		**Effect size**	
**Study**	**N (% female); mean age (SD), Education level, MSSE/3MSE**	**Language measure, dementia diagnosis, severity, and type**	**% with dementia ML/BL % no dementia ML/BL**	**RR, OR, HR**
Hack et al., 2019	325 (100%); 75+ years (NA), Grade school (*n* = 15), High school (*n* = 14), Bachelor’s degree (*n* = 123), Master’s degree or higher (*n* = 173), NA	Questionnaire developed by the School Sisters of Notre Dame DSM-IV, ADLs, CERAD, MMSE, Delayed Word Recall, Verbal Fluency, Boston Naming, Constructional Praxis	ML: 27/109; BL: 82/109 ML: 60/216; BL: 156/216	Bilingualism was not associated with a reduced risk of dementia (OR: 1.17, 95% CI: 0.69, 1.98)
Lawton et al., 2015	81 (64%) Baseline ML: 4.99 (4.17), 3MSE 78.87 (9.90) BL:7.70 (4.88), 3MSE 79.56 (15.57) Follow-up ML: age 81.10 BL: age 79.31	ARSMA-II	ML: 54/1154 BL: 27/624	BL did not decrease the risk of dementia *p* = .72, AD *p* = .59, or VaD *p* = .53
Ljungberg et al., 2016	818 (51%) 73.6 (8.9) Baseline ML: 73.8 (9.0), 6.9 (1.5), 26.6 (2.3) BL: 65.7 (6.6), 14.2 (4.3), 28.7 (1.7) Follow-up ML: 78.1 (6.1), 6.5 (1.6), 25.3 (2.3) BL: 76.0 (7.7), 12.0 (2.4), 26.8 (1.6)	Language History Questionnaire DSM-IV, NINCDS–ADRDA AD, VaD, LBD, FLD, PD, and UD	ML: 102 (13.86%), 634 (86.14%) BL: 10 (12.2%), 72 (87.8)	BL did not decrease risk of dementia (*p* = .50) or AD (*p* = .36), even after adjusting for age and sex (*p* = .29)
Yeung et al., 2014	ML: 576 (61.6%), 76.1 (6.2), 10.7 (2.8), 3MS 91.2 (5.7) BL: 54 (70.4%), 75.5 (5.6), 12.4 (4), 91.1 (5.6) ESL: 360 (60.6%), 75.7 (6.4), 8.7 (3.5), 87.4 (6.9)	Self-report ML: Dementia 9.4%. 3MS 91.2 (5.7) BL: Dementia 11.1, 3MS 91.1 (5.6) ESL: Dementia 9.7%, 3MS 87.4 (6.9)	ML 54 (9.4%), 492 (85.4%) BL 6 (11.1%), 46 (85.2%) ESL 35 (9.7%), 285 (79.2%)	Model 1: 1.06 (0.69, 1.63) Model 2: .13 (0.73, 1.79) Model 3: 7 (0.67, 1.72) Model 4: (0.61, 1.59) Time 1 3MS, Time 2 3MS, and Change in the 3MS: Unadjusted model, English bilingual: Time 1, 0.6 (−1.8, 2.9), Time 2, 2.5 (−0.7, 5.7), Changed in 3MS, −1.7 (−4.2, 0.8)
Zahodne et al., 2014	ML: 637 (72%), 75.66 (5.79), 5.05 (3.61) BL: 430 (64%), 74.78 (5.66), 8.30 (4.22)	Self-report (four-point Likert-type) DSM-III Probable and possible AD, VaD, LBD, and other dementias	ML: 198/637 BL: 86/344	Better self-rated bilingualism was associated with lower odds of dementia conversion. Each point on the self-report scale was associated with 0.291 lower log odds of conversion to dementia

SD: Standard Deviation; CI: Confidence Intervals; ML: Monolinguals; BL: Bilinguals; ESL: English as a Second Language; ADLs: Activities of Daily Living; CERAD: Consortium to Establish a Registry for Alzheimer’s Disease; AD: Alzheimer’s Disease; VaD: Vascular Dementia; FLD: Frontal Lobe Dementia; PD: Parkinson’s Disease; DLB: Dementia with Lewy bodies; UD: Unspecified dementia; SNAP: Suspected Non-Alzheimer Pathophysiology; MMSE: Mini-Mental State Examination; 3MS: Modified Mini-Mental State; DSM-IV: Diagnostic and Statistical Manual of Mental Disorders Version Four; (ADDTC) NINCDS-ADRDA: (Alzheimer Disease Diagnostic and Treatment Centers) National Institute of Neurological and Communicative Disorders and Stroke and the Alzheimer’s Disease and Related Disorders Association; ICD-10: International Statistical Classification of Diseases and Related Health Problems; CDR: Clinical Dementia Rating; DSM-III: Diagnostic and Statistical Manual of Mental Disorders, Third Edition; RR: Relative Risk; OR: Odds Ratios; HR: Hazard Ratios; NA: Not Available

### Description of Mild Cognitive Impairment and Dementia

Years before clinical diagnosis of a dementia-related disorder, an individual may experience MCI which can either be of the amnestic or non-amnestic type (Pandya et al., 2016). The former is marked by memory impartment more severe than would be expected for the age of the individual and this is a risk factor for AD. In the non-amnestic type, other cognitive abilities (e.g., language) rather than memory are affected and this is a risk factor mainly for other types of dementia. However, some individuals who experience MCI of either type do not necessarily progress to AD or other forms of dementia (Pandya et al., 2016). Dementia is a progressive clinical syndrome presenting with impairment in cognition, daily functioning, and changes in behavior in the absence of any impairment in consciousness (Vinters, 2015). While dementia is an umbrella that describes a significant cognitive and functional decline usually caused by a wide range of neurodegenerative diseases, AD has a specific etiology marked by a progressive and irreversible amnestic disorder followed by a decline in other cognitive abilities and behavior as well as neuropsychiatric dysfunctions resulting in total dependence (Vinters, 2015). Diagnosis of AD is based on clinical presentation (e.g., Diagnostic and Statistical Manual of Mental Disorders) and neuropsychological assessment while neuroimaging is used to support clinical evaluation. However, a definite diagnosis can only be given by the NINCDS-ADRDA criteria with histopathological evidence supporting clinical diagnosis (Dubois et al., 2007).

### Data Extraction and Risk of Bias

We extracted information on sample size, sex, mean age at diagnosis, education level, language measure, measures to diagnose dementia, dementia subtype, degree of cognitive impairment outcomes, and study results. Two authors (SB and MF) independently assessed risk of bias at both the study and outcome level by using the modified version of the Newcastle-Ottawa Scale (NOS) to assess risk of bias for cross-sectional studies (Wells et al., 2015). The modified NOS allows to allocate a maximum of 10 stars to each study across three domains: selection of study groups (range 0–5), comparability of study groups (range 0–2), and exposure/outcome ascertainment (range 0–3). For longitudinal studies, we used the original version of the NOS for cohort studies, which allows allocating a maximum of nine stars across the same domains as in the modified version.

Because the included studies were sufficiently similar regarding the research question, methodology, and outcome, we conducted a quantitative synthesis of the data by meta-analyzing effect sizes from included studies.

In cross-sectional studies, the authors reported the age of symptom onset for AD and age of clinical diagnosis for MCI, dementia, and AD as absolute numbers in years. Longitudinal prospective studies reported the risk of dementia as relative risk – the risk of developing dementia in bilinguals relative to monolingual controls and odds ratio – the odds of developing dementia given language status (i.e., bilingualism vs. monolingualism). One longitudinal prospective study reported the proportional hazard ratios in estimating the relationship between early foreign language instruction and the risk of developing MCI later in life (Wilson et al., 2015).

### Data Analysis

Our primary outcome measures were the age of symptom onset and age at diagnosis of MCI or dementia and the risk of developing dementia. A secondary outcome included the degree of disease severity at dementia diagnosis. Here, age at diagnosis was defined as *the age at which participants were diagnosed with MCI, AD, or dementia* and age of symptom onset was defined as *the participants’ or informants’ retrospective recall of the age at which the first symptoms of cognitive impairment started*. However, most studies that reported the age of symptom onset included participants with AD, not dementia. Therefore, we could only conduct a meta-analysis on the age of symptom onset for participants with AD, not dementia. Our secondary outcome was the degree of cognitive impairment as measured by the Mini-Mental State Examination (MMSE; Folstein, Folstein, & McHugh, 1975) during dementia or AD diagnosis, and dementia risk.

All the meta-analyses conducted here were based on random-effects models at an alpha level of .05 with the Knapp-Hartung adjustment (IntHout, Ioannidis, & Borm, 2014). Because studies did not provide individual-level data, we retrieved summary data. One study included two monolingual groups: one Mandarin and one Cantonese (Zheng et al., 2018). To increase the sample size, we combined the sample sizes, means, and standard deviations on the age of symptom onset, age of clinical diagnosis, and degree of cognitive impairment from the Mandarin and Cantonese group to form one monolingual group. For these calculations, we used the formula provided by the Cochrane Collaboration (Higgins & Green, 2016). For degree of cognitive impairment at dementia diagnosis, we presented MMSE scores (range: 0–30) as Hedges’ *g* between monolinguals and bilinguals because one study (Lawton et al., 2015) reported scores from the Modified Mini-Mental Status Examination (3MSE), which uses a scale from 0 to 100 points. Also, as not all prospective studies provided the same outcome results (one study provided hazard ratios and others provided log odds ratios), we extracted the unadjusted raw values of participants who had remained free of dementia and of those who had converted to dementia from the mono- and bilingual group. These values are unadjusted values but it was necessary to use these in order to combine results into a meta-analysis.

Data were analyzed using Comprehensive Meta-Analysis Software: version 3 (Borenstein, Rothstein, & Cohen, 2005). For cross-sectional studies, we presented mean differences between monolinguals and bilinguals for our primary and secondary outcomes (mean age in years and mean MMSE scores). For longitudinal studies, we presented odds ratio. We presented 95% confidence intervals (CI) around the pooled estimates (Riley, Higgins, & Deeks, 2011). We also computed 95% prediction intervals (PI), which reflect the distribution of effect sizes across different settings and estimate the expected effect sizes for future settings (IntHout, Ioannidis, Rovers, & Goeman, 2016). However, we computed PIs for meta-analyses with at least 10 studies (Hedges & Vevea, 1998). We used tau-squared (*T*^*2*^) to investigate between-study heterogeneity, with a non-zero *T*^*2*^ value indicating between-study heterogeneity. To investigate small-study effects, we generated funnel plots for meta-analyses that include at least 10 studies (Lau, Ioannidis, Terrin, Schmid, & Olkin, 2006). To explore the impact that imputing missing studies might have on the pooled estimate, we conducted Duval and Tweedie’s trim and fill test (Duval & Tweedie, 2000). We did not conduct formal tests for funnel plot asymmetry in meta-analyses with fewer than 10 studies (Sterne et al., 2011).

Because we did not pre-specify potential covariates and to avoid data dredging (Thompson & Higgins, 2002), we restricted our investigation of heterogeneity to immigration status (Fuller-Thomson & Kuh, 2014; Mukadam et al., 2017) and dementia etiology due to its clinical relevance (Bialystok, Abutalebi, Bak, Burke, & Kroll, 2016; IntHout et al., 2016). In two subgroup meta-analyses, we compared studies that had recruited participants with dementia (irrespective of etiology) to studies that had recruited participants with AD (specific etiology) on the age of dementia and AD diagnosis. In the other subgroup meta-analysis, we compared studies explicitly mentioning that the statistical analyses had been adjusted for immigration status or at least that the analytic cohort did not include migrants to studies not explicitly mentioning whether the statistical analyses had adjusted for migration status or whether the analytic sample had included migrants. We reported the pooled estimates for heterogeneity in subgroup meta-analyses.

## Results

### Data Collection Process

We extracted demographic data including sample size, percentage of females in each group, and education level. Moreover, we extracted methodological data including the operationalization and measurement of participants’ language profiles, type of diagnosis (i.e., MCI, dementia, or AD), as well as the measurement tools used for making the clinical diagnosis of MCI or any dementia. We also extracted data for each outcome in each group including mean age of dementia diagnosis, mean age of dementia symptom onset, risk of MCI or dementia, and degree of cognitive impairment. We were able to extract sufficient data on age of MCI (*k* = 4) and dementia clinical diagnosis (*k* = 13), AD symptom onset (*k* = 7), degree of cognitive impairment (*k* = 12), and risk of dementia (*k* = 5) to conduct a meta-analysis on each of these outcomes. The total number of participants in cross-sectional studies was 4671 including 2376 monolinguals and 2295 bilinguals (Table 1 and 2). There were 121 monolinguals and 159 bilinguals in cross-sectional studies with MCI diagnosis as an outcome (Table 1), and 2256 monolinguals and 2136 bilinguals in studies with dementia diagnosis as an outcome (Table 2). There were six longitudinal prospective studies comprising a total of 4227 participants (Tables 3 and 4).

### Study Characteristics

The operationalization of bilingualism differed across studies including: “had spent the majority of their lives, at least from early adulthood regularly using at least two languages” (Bialystok et al., 2007; Craik, Bialystok, & Freedman, 2010), “the ability to communicate in two or more languages in interaction with other speakers of these same languages” (Alladi et al., 2013; Alladi et al., 2017), “individuals had spent the majority of their lives, beginning at least in early adulthood, speaking two or more languages fluently—ideally daily, but at least weekly” (Bialystok et al., 2014; Chertkow et al., 2010; Ossher, Bialystok, Craik, Murphy, & Troyer, 2012), “able to communicate fluently at least in 2 languages and made regular use for both” (Estanga et al., 2017), “ability to meet the communicative demands of the self and the society in their normal functioning in 2 or more languages in their interaction with other speakers of any or all of these languages” (Ramakrishnan et al., 2017), “fluent in a second language and had used both languages consistently throughout most of his or, her life” (Schweizer, Ware, Fischer, Craik, & Bialystok, 2012), “determined on the basis of second language proficiency and frequency of use” (Woumans et al., 2015) or did not apply a specific definition (Lawton et al., 2015; Ljungberg et al., 2016; Perani et al., 2017; Wilson et al., 2015; Yeung et al., 2014; Zahodne et al., 2014). One study used more strict definitions for monolingualism and bilingualism including “speaking English for all or most of one’s life and being fluent in English, but not in any other language” and “speaking both Welsh and English for all or most of one’s life and being fluent in both languages, but not in any other languages”, respectively (Clare et al., 2016).

Studies used different types of measurements for bilingualism (Tables 1, 2, 3, and 4). While several cross-sectional studies used validated measures including questionnaires to measure bilingualism (Bialystok et al., 2014; Clare et al., 2016; Estanga et al., 2017; Lawton et al., 2015; Ossher et al., 2012), others used non-validated methods (Alladi et al., 2013; Bialystok et al., 2007; Chertkow et al., 2010; Schweizer et al., 2012; Woumans et al., 2015), or did not report the method of collection (Craik et al., 2010; Ramakrishnan et al., 2017). Similarly, one longitudinal study assessed participants’ language profiles with a non-validated measure (Wilson et al., 2015), two used a questionnaire but did not report their psychometric properties (Hack et al., 2019; Ljungberg et al., 2016), while one study validated their measure as part of the study (Zahodne et al., 2014).

There were differences in the type of MCI and dementia across studies (Table 1–4). Four studies recruited participants with MCI (Bialystok et al., 2014; Ossher et al., 2012; Ramakrishnan et al., 2017; Wilson et al., 2015). The type of MCI differed across studies with two studies recruiting individuals with MCI without describing its subtype (Bialystok et al., 2014; Wilson et al., 2015), another study recruited individuals with single and multiple domain amnestic MCI (Ossher et al., 2012), while still another study recruited individuals with amnestic and non-amnestic MCI (Ramakrishnan et al., 2017).

The tools for diagnosing MCI and dementia as well as the dementia subtypes differed across studies (Table 1–4). For MCI, studies either did not report the method of diagnosis (Bialystok et al., 2014), diagnosed MCI during a clinical interview with neuropsychological tests (Ossher et al., 2012), adopted the Mayo Clinic MCI criteria [(Ramakrishnan et al., 2017) Table 1], or the NINCDS-ADRDA criteria (Wilson et al., 2015). The diagnosis of dementia was often based on a clinical interview conducted by medical staff (e.g., a neurologist) and a neuropsychological assessment and using the NINCDS-ADRDA criteria (Bialystok et al., 2007; Chertkow et al., 2010; Lawton et al., 2015; Ljungberg et al., 2016), the International Classification of Diseases 10 (Clare et al., 2016), or the Diagnostic and Statistical Manual of Mental Disorders, 4th Edition (Alladi et al., 2013), among others (Table 2).

Studies recruited participants with a wide range of dementia subtypes including the behavioral variant frontotemporal dementia, progressive non-fluent aphasia, semantic dementia, frontotemporal dementia-motor neuron disease, corticobasal degeneration, and progressive supranuclear palsy (Alladi et al., 2017), AD (Clare et al., 2016; Ljungberg et al., 2016; Woumans et al., 2015), vascular dementia (Alladi et al., 2013; Ljungberg et al., 2016; Zahodne et al., 2014), mixed AD with cardiovascular disease, frontotemporal dementia, dementia with Lewy bodies (Alladi et al., 2013; Zahodne et al., 2014), probable AD (Bialystok et al., 2014; Chertkow et al., 2010; Craik et al., 2010; Lawton et al., 2015; Perani et al., 2017; Schweizer et al., 2012; Zahodne et al., 2014), possible AD (Bialystok et al., 2007; Lawton et al., 2015; Zahodne et al., 2014), dementia due to other neurodegenerative disorders, cardiovascular disease (Bialystok et al., 2007), preclinical AD (Estanga et al., 2017), frontal lobe dementia (Ljungberg et al., 2016), and dementia [(Hack et al., 2019; Yeung et al., 2014) Table 2].

### Risk of Bias for Cross-Sectional Studies

Risk of bias for cross-sectional studies is presented in Table 5. Most cross-sectional studies employed acceptable sampling methods (*k* = 16; 100%), but most did not provide evidence for power calculations (*k* = 14; 88%). While some studies (*k* = 5; 31%) administered a validated measure of language ability, half of all studies (*k* = 8; 50%) used non-validated measures, including self- or proxy-reported measures (e.g., family member) or did not report the method of data collection (*k* = 2, 13%). Some studies (*k* = 14, 88%) controlled for important covariates such as immigration status and education either methodologically or statistically while others did not control for any covariates (*k* = 2, 13%).

**Table 5:** Risk of bias for cross-sectional studies.

**Study**	**Selection**				**Comparability**		**Outcome**		**Total/10**
	Representativeness of the sample	Sample size calculation	Non-respondents	Ascertainment of exposure	Controls for most important factor	Controls for any additional factor	Assessment of the outcome	Statistical test	
Alladi 2017	**–**	**–**	★	**–**	★	★	★	★	5
Alladi 2013	**–**	**–**	★	**–**	**–**	★	★	★	4
Bialystok 2014	**–**	**–**	**–**	★	★	★	★	★	5
Bialystok 2007	**–**	**–**	★	★	★	★	★	★	6
Chertkow 2010	**–**	**–**	★	★	★	★	★	★	6
Craik 2010	**–**	**–**	★	**–**	**–**	★	★	★	4
Clare et al., 2016	**–**	★	★	★	★	★	★	★	7
Estanga 2016	**–**	**–**	★	★	★	★	★	★	6
Lawton 2015	**–**	**–**	★	★	★	★	★	**–**	5
Ossher 2013	**–**	**–**	**–**	**–**	★	★	★	★	4
Perani 2017	★	**–**	**–**	★	★	**–**	★	★	5
Ramakrishnan 2017	**–**	**–**	★	**–**	★	★	★	★	5
Schweizer 2012	**–**	**–**	**–**	**–**	★	★	★	★	4
Woumans 2015	**–**	**–**	★	**–**	★	★	★	★	5
Yeung 2014	**–**	★	★	**–**	★	★	★	★	6
Zheng 2018	**–**	**–**	★	★	★	★	★	★	6

A maximum of 10 stars can be given to each study

### Risk of Bias for Longitudinal Studies

Risk of bias for longitudinal studies is presented in Table 6. All longitudinal studies employed poor sampling methods and either administered a language questionnaire, of which there was no mention of the psychometric properties, or they relied on self-report during a structured interview. Potential confounding factors including age, sex, and apolipoprotein E (*APOE*) ε4 allele status (Ljungberg et al., 2016); age, sex, and years of formal education (Wilson et al., 2015); age, sex, education, and subjective memory loss (Yeung et al., 2014); country of origin, gender, education, time spent in the current home country (United States of America), recruitment wave, and age at enrollment (Zahodne et al., 2014); occupation, education, baseline age, immigration status, *APOE* ε4 allele status, idea density, and grammatical complexity (Hack et al., 2019) were controlled for. Finally, all studies had adequate assessments of the outcome including blind assessments for dementia diagnosis and appropriate follow-up periods, as well as reported sufficient information on attrition rate.

**Table 6:** Risk of bias for longitudinal prospective studies.

**Study**	**Selection**			**Comparability**			**Outcome**			**Total/9**
	Representativeness of the exposed cohort	Selection of the non-exposed cohort	Ascertainment of exposure	Demonstration that outcome of interest was not present at start of study	Controls for most important factor	Controls for any additional factor	Assessment of outcome	Was follow-up long enough for outcomes to occur	Adequacy of follow up of cohorts	
Hack 2019	**–**	**–**	**–**	★	★	★	★	★	★	6
Ljungberg 2016	**–**	**–**	**–**	★	★	★	★	★	★	6
Wilson 2015	**–**	**–**	**–**	★	★	★	★	★	★	6
Yeung 2014	**–**	**–**	**–**	★	**–**	**–**	★	★	**–**	3
Zahodne 2014	**–**	**–**	★	**–**	★	★	★	★	**–**	5

A maximum of 9 stars can be given to each study

### Meta-Analyses of Cross-Sectional Studies: Age of Symptom Onset, Diagnosis and Disease Severity at Dementia Diagnosis

#### Age at Alzheimer’s Disease Symptom Onset

**Fig. 2:**
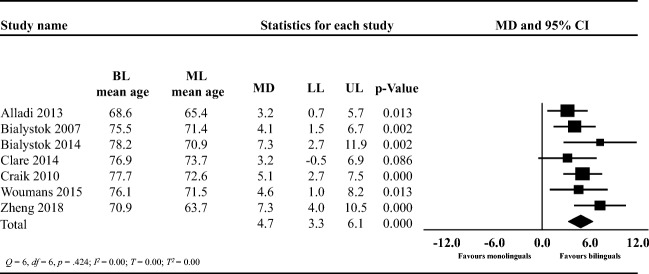
Forest plot showing the mean difference (MD) in the age of Alzheimer's Disease symptom onset between bilinguals (BL) and monolinguals (ML); lower limit (LL), upper limit (UL); CI: confidence intervals

The mean difference between mono- and bilinguals at the age of AD symptom onset was 4.7 years (95% CI: 3.3, 6.1; Fig. 2). The *t* value was 8.06 with a two-tailed *p <* 0.001. Therefore, bilinguals were significantly older than monolinguals at the time of AD symptom onset. The *Q*-value was 6 with 6 *df* and with *p* = 0.424. Also, *I*^*2*^ was 0.00 and the variance in true effect sizes was *T*^*2*^ = 0.00, with *T* = 0.00.

#### Age at MCI and Dementia Diagnosis

**Fig. 3:**
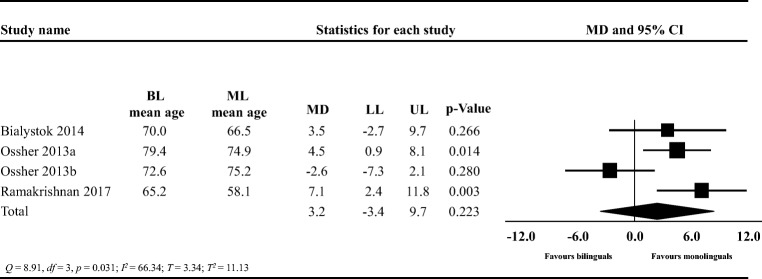
Forest plot showing the mean difference (MD) in the age of mild cognitive impairment diagnosis between bilinguals (BL) and monolinguals (ML); LL: lower limit, UP: upper limit; CI: confidence intervals

**Fig. 4:**
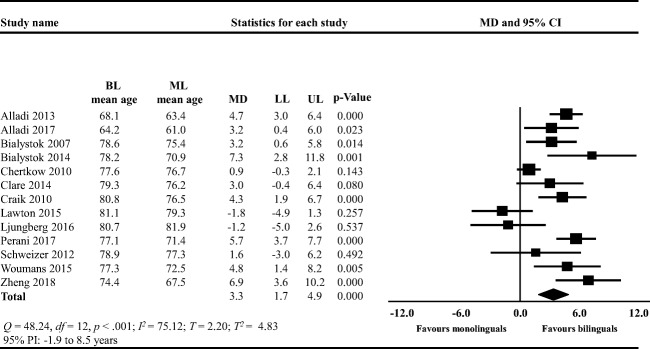
Forest plot showing the mean difference (MD) in the age of dementia diagnosis between bilinguals (BL) and monolinguals (ML); LL: lower limit, UP: upper limit; CI: confidence intervals

Bilinguals were on average 3.2 years (95% CI: −3.4, 9.7; Fig. 3) older than monolinguals at MCI diagnosis. This mean difference was not statistically significant (*t* = 1.53, two-tailed *p* = .223). There was evidence that studies did not share a common effect size but that the true effects varied (*Q* = 8.91, *df* = 3, *p* = .031). Approximately 66% of the observed variance reflected the difference in true effect sizes rather than sampling error (*I*^*2*^ = 66.34). The variance in true effect sizes was *T*^*2*^ = 11.13, with *T* = 3.34. Bilinguals were on average 3.3 years (95% CI: 1.7, 4.9; Fig. 4) older than monolinguals at dementia diagnosis. This mean difference was statistically significant (*t* = 4.3, two-tailed *p* < .001). There was evidence that studies in this analysis likely did not share a common effect size but that the true effects varied (*Q* = 48.24, *df* = 12, *p* < .001). The *I*^*2*^ was 75.12 indicating that approximately 75% of the observed variance reflected the difference in true effect sizes rather than sampling error. The variance in true effect sizes was *T*^*2*^ = 4.83, with *T* = 2.20. The 95% PIs ranged from −1.9 to 8.5 years. Overall, in this analysis, we observed a high degree of heterogeneity.

#### Subgroup Analysis: Type of Diagnosis (Dementia vs. AD)

**Fig. 5:**
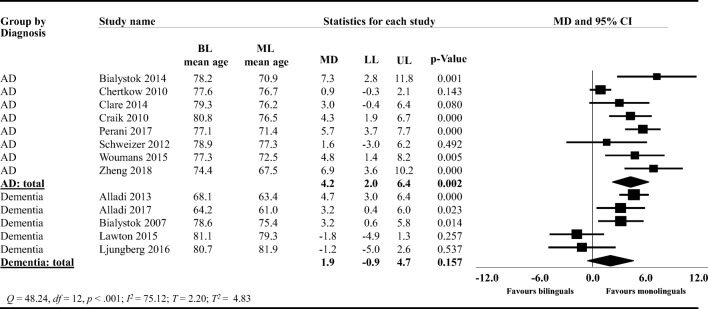
Forest plot showing the mean difference (MD) in the subgroup meta-analysis comparing studies including participants with AD to studies including participants with dementia on the age of AD and dementia diagnosis between bilinguals (BL) and monolinguals (ML); AD: Alzheimer’s disease; LL: lower limit, UP: upper limit; CI: confidence intervals

We conducted a post hoc subgroup analysis to explore the source for this heterogeneity. We compared studies including participants with AD to studies including participants with dementia. Bilinguals in the AD subgroup (*k* = 8; Fig. 5) were on average 4.2 years (95% CI: 2.0, 6.4) significantly older than monolinguals (*t* = 4.13, two-tailed *p* = .002). Bilinguals in the dementia subgroup (*k* = 5; Fig. 5) were on average 1.9 years (95% CI: −0.9, 4.7) older than monolinguals, but this between-group difference was not statistically significant (*t* = 1.52, two-tailed *p* = .157). We also compared the mean difference for the AD and dementia subgroups to explore whether there were any significant differences between the two subgroups (Fig. 5). The mean difference in years (2.3, 95% CI: −5.9, 1.2) between the two subgroups was not statistically different (*F* = 2.04, *df* = 1, 11, two-tailed *p* = 0.181). The pooled estimates for heterogeneity in this subgroup analysis were *T*^2^ = 4.83, *T* = 2.20, *I*^2^ = 75.12, *Q* = 48.24, with *df* = 12, and *p* < .001.

#### Subgroup analysis: immigration status (adjusted vs. did not adjust for immigration)

**Fig. 6:**
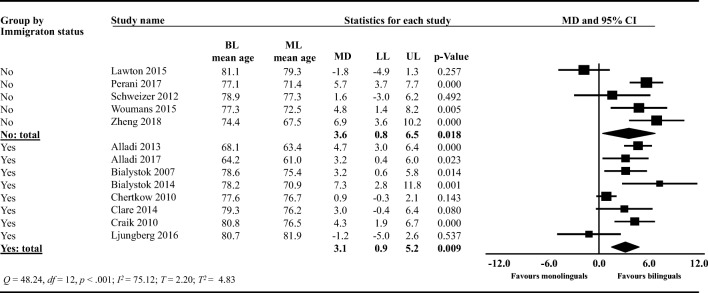
Forest plot showing the mean difference (MD) in the subgroup meta-analysis comparing studies that had adjusted for immigrations status to studies that had not adjusted for immigration status on the age of dementia diagnosis between bilinguals (BL) and monolinguals (ML); LL: lower limit, UP: upper limit; CI: confidence intervals. Studies that had not adjusted for immigration status are categorized as *No* and studies that had adjusted for immigration status are categorized as *Yes*.

We conducted a *post-hoc* subgroup analysis (Fig. 6) exploring whether immigration status was a potential source of heterogeneity. Bilinguals in studies adjusting for immigration status (*k* = 8) were on average 3.1 years (95% CI: 0.9, 5.2) older than monolinguals at dementia diagnosis (*t* = 3.17, two-tailed *p* = .009). In studies that did not adjust for immigration status (*k* = 5), bilinguals were on average 3.6 years (95% CI: 0.8, 6.5) older than monolinguals at dementia diagnosis (*t* = 2.97; two-tailed *p* = .018). The mean difference in years (0.5, 95% CI: −4.1, 3.0) between the two subgroups was not statistically different (*F* = 0.10, *df* = 1, 11, two-tailed *p* = 0.752). The pooled estimates for heterogeneity in this subgroup analysis were *T*^2^ = 4.83, *T* = 2.20, *I*^2^ = 75.12, *Q* = 48.24, with *df* = 12, and *p* < .001.

#### Disease Severity

**Fig. 7:**
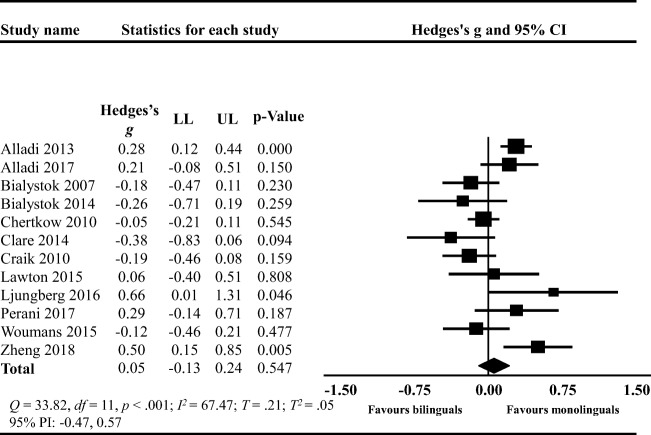
Forest plot showing the standardized mean difference (Hedges’s *g*) in the degree of disease severity at dementia diagnosis between bilinguals (BL) and monolinguals (ML); LL: lower limit, UP: upper limit; CI: confidence intervals

There was no significant difference between mono- and bilinguals in disease severity at the age of dementia diagnosis (Hedges’ *g =* 0.05; 95% CI: −0.13, 0.24; *t* = 0.62, two-tailed *p* = .547; Fig. 7). The *Q*-value was 33.82 with *df* = 11 and *p* < .001. Approximately 67% (*I*^2^) of the observed variance reflected the difference in true effect sizes rather than sampling error. The variance in true effect sizes was *T*^*2*^ = .05 and *T* = .21. The PIs ranged between −0.47 and 0.57 MMSE points.

### Meta-Analysis of Longitudinal Prospective Studies: Risk of Dementia

**Fig. 8:**
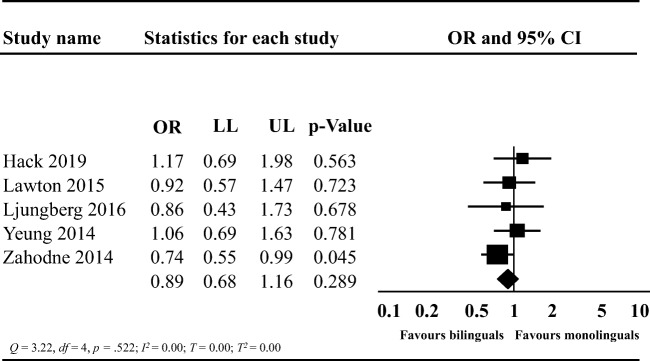
Forest plot showing the odds of developing dementia between monolinguals and bilinguals; OR: odds ratio; LL: lower limit, UP: upper limit; CI: confidence intervals

We performed a meta-analysis on longitudinal prospective studies (Fig. 8). Results from this meta-analysis (*k* = 5) showed that bilingualism was not associated with a reduction in the risk of dementia (OR: 0.89; 95% CI: 0.68, 1.16, *t* = −1.22, two-tailed *p* = 0.289) when compared to monolingualism. There was no evidence of heterogeneity (*Q* = 3.22, *df* = 4, *p* = .522; *I*^*2*^ = 0.00; *T*^*2*^ = 0.00; *T* = 0.00).

### Small-Study Effects

**Fig. 9:**
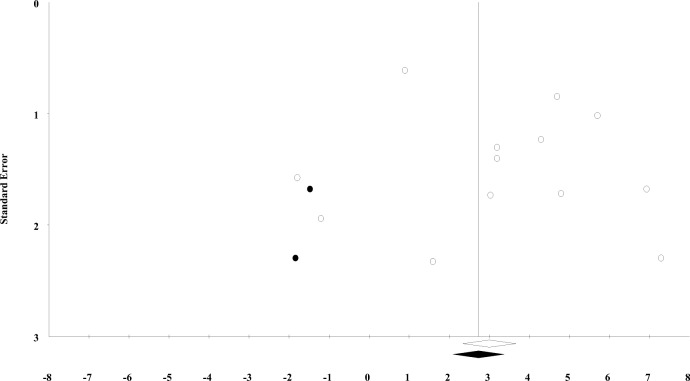
Funnel plot showing standard error by difference in means with observed (white dots) and imputed estimates (black dots) for the meta-analysis including dementia as the outcome

**Fig. 10:**
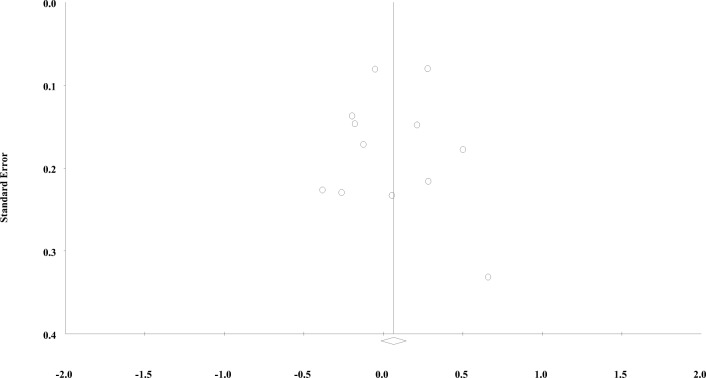
Funnel plot showing standard error by difference in standardized means (Hedges’s *g*) with observed estimates for the meta-analysis including disease severity at dementia diagnosis as the outcome

To address small-study effects, we generated funnel plots (Figs. 9 and 10). One funnel plot (Fig. 9) shows the observed (white dots) and imputed (black dots) effect sizes. Here, visual inspection showed that the observed data points tend to cluster on the right-hand side of the funnel plot indicating a minor asymmetry, suggesting the presence of small-study effects. However, Egger’s test was not significant with an intercept of 1.03 and CIs including −2.15 and 4.20 and with a *t* value of 0.71, *df* = 11, and a 1-tailed *p* value of 0.246. The Duval and Tweedie’s Trim and Fill test showed that the adjusted effect size (black diamond) would be 2.7 (95% CIs 1.3, 4.1) if the imputed studies had been included in the analysis. This indicates that even the adjusted effect size remained statistically significant.

Visual inspection of the second funnel plot (Fig. 9) for the meta-analysis on disease severity (Fig. 10) showed a slight asymmetry on the right-hand side of the plot indicating a minor asymmetry, suggesting the presence of small-study effects. However, Egger’s test was not significant (one-tailed *p* value of 0.420) with an intercept of −0.267 (95% CI: −3.158, 2.623) and a *t-*value of 0.21 with *df* = 11. The Duval and Tweedie’s Trim and Fill showed that the adjusted effect size (black diamond) would be 0.05 (95% CI: −0.10, 0.21) if the imputed studies had been included in the analysis. Even in the likelihood of small-study effects or publication bias (De Bruin, Treccani, & Della Sala, 2015), the adjusted effect size remained similar to the observed effect size.

## Discussion

While some studies have linked bilingualism to a delay in AD symptom onset and dementia diagnosis (Perani et al., 2017; Perquin et al., 2013; Schweizer et al., 2012), others have not reported such benefits (Mukadam et al., 2017). Some authors have argued that education and immigration status, among other confounders, may influence the relationship between bilingualism and dementia in cross-sectional studies (Mukadam et al., 2017). As such, further research is needed (Del Maschio et al., 2018; Grundy & Anderson, 2017; Woumans, Versijpt, Sieben, Santens, & Duyck, 2017).

### Mild Cognitive Impairment

Meta-analytic results did not suggest that bilingualism delays the diagnosis of MCI. Due to the small number of included studies (*k* = 4) and small sample sizes (monolinguals *n* = 131; bilinguals *n* = 169), it is likely that this meta-analysis was underpowered and consequently, a type II error is possible (Hedges & Pigott, 2001). Studies had recruited participants with different types of MCI including single-domain amnestic and multiple-domain amnestic MCI (Ossher et al., 2012), amnestic and non-amnestic MCI (Ramakrishnan et al., 2017), or did not specify the subtype (Bialystok et al., 2014). Given the low number of included studies in this meta-analysis, we could not conduct a subgroup analysis to explore whether bilingualism was associated with a delayed diagnosis of MCI in relationship to the different subtypes of MCI. Of note, while MCI is a risk factor for dementia and AD, not all individuals with MCI will progress to AD or dementia (Albert et al., 2011). Therefore, the putative beneficial effects of bilingualism may be more salient at the beginning of the AD clinical spectrum rather than in milder forms of cognitive impairment such as MCI. Notably, a longitudinal study showed that foreign language instruction during childhood and adolescence lowered the risk of non-amnestic MCI but not amnestic MCI (Wilson et al., 2015), which supports some of the primary cross-sectional studies (Bialystok et al., 2014; Ossher et al., 2012; Ramakrishnan et al., 2017).

### Age of AD Symptom Onset

Our meta-analysis showed that bilinguals experienced AD symptoms on average 4.7 years later than monolinguals. While we did not observe significant heterogeneity, given the low number of studies (*k* = 7), caution in interpreting these findings as homogenous is warranted (Ioannidis, Patsopoulos, & Evangelou, 2007). These findings are in line with previous studies which show that speaking multiple languages is associated with better cognitive health in old age (Ihle, Oris, Fagot, & Kliegel, 2016; Kavé, Eyal, Shorek, & Cohen-Mansfield, 2008). Notably, the included studies did not provide a comprehensive profile of participants’ spoken languages, and because of this, we could not further investigate whether second-language proficiency, frequency of use, and age of acquisition played a moderating role in the observed delay in AD symptom onset (Del Maschio et al., 2018). When assessing AD symptom onset, researchers asked participants to retrospectively recall the age at which participants first began noticing AD symptoms. However, participants’ recall is often inaccurate and recall bias might have distorted participants’ reported estimates questioning its accuracy (Van den Bergh & Walentynowicz, 2016). In this meta-analysis (Fig. 2) studies tended to have small sample sizes (*N* median: 68.5) questioning the precision of the observed effect sizes (Cumming, 2014). Consequently, whether the estimate is close to the true value in this meta-analysis remains uncertain.

### Age of Dementia and AD Diagnosis

Bilinguals were diagnosed with dementia on average 3.3 years later than monolinguals. According to the 95% PI (−1.9 to 8.5), we could expect that in some 95% of all populations comparable to those in this meta-analysis (Fig. 4), the association between bilingualism and dementia may be strong, while in others, this association may be absent or may even tend to be in the opposite direction (Riley et al., 2011). Therefore, the beneficial association between bilingualism and delayed dementia diagnosis may appear only in some populations. While there are several possible explanations for wide PIs such as high risk of bias, we explored whether clinical differences across studies may be associated with the magnitude of the observed effect size in the meta-analysis in Fig. 4 (Sterne et al., 2011; Thompson, 1994). To address this, we conducted a post hoc subgroup analysis (Borenstein & Higgins, 2013; Oxman & Guyatt, 1992) comparing studies including participants with dementia (irrespective of etiology) to studies including participants with AD (specific etiology). In this analysis, bilinguals were not older than monolinguals at dementia diagnosis (mean difference: 1.9 years) but were 4.2 years older at AD diagnosis. Here, the between-subgroup mean difference did not differ. Low statistical power, as indicated by wide CI, the low number of studies per subgroup (dementia: *k* = 5; AD: *k* = 8), and a low sample sizes per study might explain the lack of difference in the dementia subgroup and in the between-subgroup analysis (Riley et al., 2011).

However, subgroup analyses are by default observational and because of this, we cannot be certain that participants in each subgroup were similar other than in the type of diagnosis. For example, the AD subgroup might have included a large portion of participants who could speak several languages and the dementia subgroup might have included bilinguals who spoke only two languages. Therefore, while bilinguals vs. monolinguals were older at AD but not at dementia diagnosis, we cannot be certain that this was due to differences in the type of diagnosis, and that findings should only be interpreted as hypothesis-generating (Thompson & Higgins, 2002).

### Risk of Dementia

The meta-analysis including prospective studies showed no significant risk reduction in developing dementia among bilinguals compared to monolinguals. Our effect size favored bilinguals more than the effect size from the previous meta-analysis [odds ratio: 0.89; 95% CI: 0.68–1.16; (Mukadam et al., 2017)]. From our systematic review, we decided to exclude one study because it did not clearly define its control group as monolingual (Sanders et al., 2012), but it was included in the previous meta-analysis (Mukadam et al., 2017). The difference in the included studies between our and the previous meta-analysis might explain the difference in the magnitude of the effect sizes. Moreover, while results showed no risk reduction in dementia among bilinguals, the trend favoring bilinguals in our meta-analysis (Fig. 7) needs to be carefully considered. Given the low number of studies (*k* = 5), our meta-analysis might not have reached sufficient statistical power to detect a true effect (Hedges & Pigott, 2001). The CIs in each study were relatively wide indicating low statistical power and poor precision (Cumming, 2014). Therefore, each study was also likely underpowered to detect a true effect, if such an effect existed (Ioannidis, 2005, 2008). We did not find evidence of heterogeneity in this meta-analysis. Given the low number of studies in this meta-analysis, the *Q* statistic was likely underpowered, however. Notably, lack of heterogeneity does not necessarily indicate homogeneity (Ioannidis et al., 2007); interpreting a non-significant heterogeneity test in a meta-analysis with few studies is problematic (Rücker, Schwarzer, Carpenter, & Schumacher, 2008).

### Possible Mechanisms and Disease Severity at Dementia Diagnosis

Some authors have argued that while cross-sectional studies generally tend to show a later dementia diagnosis for bilinguals vs. monolinguals, these studies are more susceptible to the confounding effects of education or cultural differences (Mukadam et al., 2017). Given that our meta-analyses included studies that had adjusted for education, it is unlikely that education had confounded the observed delays in dementia and AD diagnoses among bilinguals. We also conducted a subgroup meta-analysis comparing studies that had adjusted for immigration to those that did not explicitly mention participants’ immigration status. This analysis found that bilinguals were older than monolinguals at dementia diagnosis regardless of subgroup membership. This suggests that immigration might not have played a role in delaying the age of dementia diagnosis in bilinguals relative to monolinguals in these studies. It is noteworthy to highlight that while some studies had mentioned participants’ migration status, it was occasionally problematic to discern whether authors had in fact adjusted for migration status because there was no statement explicitly addressing the analytical approach for adjusting for this variable.

Even if bilinguals were delaying seeking medical attention due to cultural differences, we would still expect them to demonstrate greater cognitive impairment than monolinguals at dementia diagnosis. However, we found no difference (Hedges’ *g* = 0.05, 95% CI: −0.13, 0.24) between mono- and bilinguals on disease severity at dementia diagnosis. This suggests that in some settings, bilingualism may be more beneficial than monolingualism to help maintain cognitive function for a longer period of time despite the presence of ongoing neuropathology (Gold, 2015). It is possible that bilingualism may help in maintaining cognitive health for a longer period of time, protecting against the impending effects of AD on cognition (Bak et al., 2014; Gold, 2015). There is evidence to suggest that bilingualism is associated with higher cognitive function in old age (Ihle et al., 2016; Kavé et al., 2008) even after adjusting for differences in intelligence levels during childhood (Bak et al., 2014). Some authors have advanced the proposition that bilingualism may enhance cognitive reserve, which refers to the ability to maintain functioning levels of cognition despite the presence of a neurodegenerative disease such as AD (Perquin et al., 2013; Stern, 2012).

Supporting findings from our meta-analysis on disease severity, behavioral data indicated that mono- and bilinguals did not significantly differ in executive functions at AD diagnosis despite bilinguals being significantly older (Bialystok et al., 2014). Computed tomography scans also revealed greater atrophy of the medial temporal lobe at AD diagnosis in bilinguals vs. monolinguals matched for disease severity and despite monolinguals having higher education and job status (Schweizer et al., 2012). The medial temporal lobe is a region particularly affected by AD (Clerx et al., 2013; Visser et al., 2002). Moreover, bilinguals showed greater cerebral hypometabolism than monolinguals, which is indicative of greater neurodegeneration, and outperformed monolinguals in short- and long-term verbal and visuospatial memory, but not in language tasks (Perani et al., 2017). Given the disagreement in the field regarding the exact underlying mechanisms of bilingualism thought to promote cognitive reserve (García-Pentón et al., 2016a, 2016b), we are currently conducting a systematic review investigating the underlying brain mechanisms of bilingualism in non-clinical and clinical individuals with MCI or dementia (Brini et al., 2018a).

Because studies did not generally measure participants’ socioeconomic status, it was not possible to examine whether this factor might have contributed to the observed delays in dementia diagnosis among bilinguals. The incidence of dementia is higher in certain ethnic minorities than in Caucasian individuals (Mehta & Yeo, 2017), suggesting that socioeconomic and cultural factors may play a role in the observed relationship between bilingualism and dementia. Researchers have extensively debated how to quantify bilingualism (Del Maschio et al., 2018; Luk & Bialystok, 2013). While studies have tended to categorize participants between mono- and bilinguals (Del Maschio et al., 2018), bilingualism is a multidimensional variable that extends on a continuum (Luk & Bialystok, 2013). For example, factors including the number of languages one can speak, age of acquisition, proficiency, and frequency of use in the second language likely interact with one another and may explain differences in the observed delay in dementia among bilinguals (Del Maschio et al., 2018). However, the studies included in our meta-analysis did not formally assess these factors (Table 5–6), and because of this, we could not examine whether the different dimensions of bilingualism (Del Maschio et al., 2018; Luk & Bialystok, 2013) contributed to the observed heterogeneity in some of our meta-analyses.

### Sources of Uncertainty and Risk of Bias in Cross-Sectional and Longitudinal Prospective Studies

From our risk of bias assessment within studies, it is clear that one major source of uncertainty concerned how representative the samples were and whether the exposure had been measured appropriately (Table 5). In cross-sectional studies, because no study formally assessed monolingualism, the extent to which participants were truly monolinguals remains unclear. This would have been an important factor to assess because exposure to foreign languages through schooling or the media is ubiquitous nowadays (Laine & Lehtonen, 2018), questioning whether the monolinguals in our included studies were in fact, truly monolinguals. Generally, bilingualism was poorly defined, measured, or did not carry a specific definition. While researchers commonly defined bilingualism as “speaking two or more languages,” they did not routinely measure additional languages. This would have been a relevant factor to measure because some studies point to a dose-response relationship (Antoniou & Wright, 2017) with increasing number of languages generating a greater delay in the onset of dementia (Alladi et al., 2013; Chertkow et al., 2010; Clare et al., 2016), protection against cognitive impairment (Perquin et al., 2013), and greater cognitive health in older individuals (Ihle et al., 2016).

The assessment of participants’ language profiles and by extension, their representativeness, was also questionable in longitudinal prospective studies (Table 6). In one study, bilingualism was not associated with reduced dementia risk but those reporting speaking a second language *very well* had a 14% lower risk of developing dementia than those who reported *not at all well* (Zahodne et al., 2014). This questions whether participants who reported speaking a second language “not at all well” should have been classed as bilinguals or monolinguals and supports the notion that participants’ language profiles should be treated as a continuous rather than a dichotomous variable (Luk & Bialystok, 2013). While other studies did not show a risk reduction in dementia among bilinguals, it is likely that they were underpowered. For example, one study included 736 monolinguals but only 82 bilinguals with 102 developing dementia in the monolingual group and 10 among bilinguals (Ljungberg et al., 2016). Furthermore, only three studies adopted a questionnaire to measure bilingualism (Hack et al., 2019; Ljungberg et al., 2016; Zahodne et al., 2014). Thus, differences in the operationalization and measurement of bilingualism, as well as relatively small sample sizes, question the internal validity of the longitudinal prospective studies (IntHout, Ioannidis, Borm, & Goeman, 2015).

Further, while some cross-sectional studies adjusted for important confounders such as education and occupation status, several studies did not specify whether they had adjusted for other likely confounders nor did they routinely report participants’ immigration status (Fuller-Thomson, 2015; Fuller-Thomson & Kuh, 2014) or levels of physical activity. Levels of physical activity may be an important factor to assess as bilingualism may benefit sedentary individuals (Brini et al., 2018b) differently than highly physically active individuals (Sterne et al., 2011). Since the majority of studies did not provide evidence of power calculations, it was unclear whether they had sufficient statistical power to detect an effect if one existed particularly when adjusting for genes (Ioannidis, 2008; Sham & Purcell, 2014). For example, bilingualism may benefit participants with the apolipoprotein E (*APOE*) ε4 allele, the main genetic risk factor for AD (Galimberti & Scarpini, 2016; Liu, Kanekiyo, Xu, & Bu, 2013), differently than those without the *APOE* ε4.

Similar to the cross-sectional studies, longitudinal prospective studies did not consider participants’ baseline risk of dementia. Although one study adjusted for the *APOE* ε4 (Ljungberg et al., 2016), which is a risk factor for AD (Brini et al., 2018b), no other prospective study considered other genetic risk factors implicated in AD (Naj, Schellenberg, & Consortium, 2017). Only 147 participants (across mono- and bilinguals) were *APOE* ε4 carriers (Ljungberg et al., 2016). Of note, whether *APOE* ε4 increases the risk of vascular dementia (Rohn, 2014), frontotemporal dementia (Verpillat et al., 2002), dementia with Lewy bodies (Lovati et al., 2010), and Parkinson’s disease (Fagan & Pihlstrøm, 2017) is unclear (Lovati et al., 2010). Therefore, adjustment for the *APOE* ε4 likely did not reach sufficient statistical power (Sham & Purcell, 2014) in this study (Ljungberg et al., 2016) and its clinical relevance to other dementia etiologies may have been limited (Lovati et al., 2010). The authors also did not analyze other variants of the *APOE* including the ε2, which may confer protection against AD (Liu et al., 2013).

### Small-Study Effects

While our funnel plots showed slight asymmetry indicating possible small-study effects (Sterne, Egger, & Smith, 2001), Egger’s tests were not significant. However, results from Egger’s test should be interpreted with caution, because in the absence of severe bias, this test has low statistical power (Sterne, Gavaghan, & Egger, 2000). One possible source of small-study effects is publication bias (Egger, Smith, Schneider, & Minder, 1997), which is prevalent in the social (Franco, Malhotra, & Simonovits, 2014) and cognitive sciences (Ioannidis, Munafo, Fusar-Poli, Nosek, & David, 2014). More notably for this systematic review, it is likely present in the field of bilingualism research, too (De Bruin et al., 2015); although others (Bialystok, Kroll, Green, MacWhinney, & Craik, 2015) have contested these findings (De Bruin et al., 2015). Therefore, despite the non-significant Egger’s tests, there are reasons to believe that publication bias may be present in this field of research. In light of this, the Duval and Tweedie’s Trim and Fill test (Duval & Tweedie, 2000) showed that after imputing the estimated missing studies, bilinguals would be on average 2.7 years (95% CI: 1.3, 4.1) older than monolinguals at the time of dementia diagnosis. Even in the likelihood of publication bias (De Bruin et al., 2015), the observed effect size in this meta-analysis (Fig. 4) would not change by a large margin.

Several of our included studies had small sample sizes, which can increase the risk of type I error (Ioannidis, 2005) and inflate the effect size (Ioannidis, 2008), which can result in funnel plot asymmetry (Sterne et al., 2011). For example, if the association between bilingualism and dementia is driven by a dose-response relationship (Alladi et al., 2013; Chertkow et al., 2010; Clare et al., 2016; Ihle et al., 2016), smaller studies with a higher portion of multilingual participants may generate greater effect sizes resulting in funnel plot asymmetry (Egger et al., 1997). Moreover, as mentioned previously, bilingualism may benefit participants who occupy a higher baseline risk of dementia (e.g., by virtue of genetic risk) differently, which could also explain funnel plot asymmetry (Sterne et al., 2011). However, because most studies did not report data on the number of spoken languages or participants’ baseline dementia risk, we could not explore whether multilingualism may have contributed to funnel plot asymmetry.

### Limitations

A limitation of our meta-analyses was the inclusion of all cross-sectional studies regardless of language status. Most studies did not precisely report how many languages were spoken by their bilingual cohorts. Therefore, from our meta-analyses, it remains unclear whether the number of languages a person can speak plays a role in delaying the onset of dementia. As noted above, however, some evidence suggests that the number of languages could play a role in the risk and delay of dementia (Chertkow et al., 2010; Clare et al., 2016). Additionally, in a subgroup meta-analysis, we compared studies that recruited participants with dementia and AD. In the dementia subgroup, however, participants were diagnosed with different forms of dementia. This is a limitation because, from this subgroup, it was not possible to discern whether bilingualism was distinctively related to different dementia etiologies. Furthermore, this subgroup analysis was likely underpowered given the small number of studies (*k* = 5) and the associated large CIs.

While the results of our meta-analyses on the age of dementia and AD diagnosis are interesting, it is crucial to stress that the observed relationship between bilingualism and dementia is not causal. Cross-sectional studies are useful when examining the relationship between two variables and help to generate hypotheses that may be further tested for causal effects in experimental studies. Particularly for this review, our risk of bias assessment uncovered several sources of uncertainty due to bias within studies. For example, several factors such as the poor measurement of bilingualism, the inclusion of varying types of dementia etiologies, and lack of control over confounding factors in several of the included studies, leave us questioning the beneficial link between bilingualism and dementia.

Most studies did not report how bilinguals had acquired the second or third language, or participants’ immigration status. This is a limitation in our meta-analyses because some participants might have acquired the second language through schooling whereas others might have acquired it due to migrating to a new country. In the former case, participants might have been diagnosed in their native language (e.g., English) whereas in the latter case, participants might have been diagnosed in their non-native language (e.g., a language other than the recipient country’s national language). As such, cultural differences (Chandra et al., 2001; Chin et al., 2011) or language barriers (Lindesay, 1998; Nielsen et al., 2011) might have contributed to the observed delays in dementia diagnosis and possibly confounded the relationship between bilingualism and age of dementia diagnosis. Since there was insufficient information regarding what language was used to provide a diagnosis of MCI or dementia among bilinguals, we could not further explore whether the language of the assessment played a role in the observed delays in any of our outcomes.

### Strengths

Unlike a previous systematic review (Mukadam et al., 2017), results from cross-sectional studies were meta-analyzed to determine whether bilingualism is associated with a delayed onset of dementia and AD. This allowed us to generate a more precise estimate of the effect size. In response to previous criticisms (Fuller-Thomson, 2015; Fuller-Thomson & Kuh, 2014; Mukadam et al., 2017), we explored whether immigration status might have been related to differences in the age of dementia diagnosis by conducting a subgroup meta-analysis. We have included more recently published studies that had not been included in the previous systematic review (Mukadam et al., 2017) and therefore, provide a more up to date review of the available literature. We also registered a study protocol a priori for this systematic review.

### Suggestions for Future Research

Given the lack of a standard definition and measurement tool for mono- and bilingualism across our included studies, it is critical for future research to improve the measurement of participants’ language profile. This could mean quantifying the spectrum of language knowledge on a continuum and by measuring proficiency, frequency of use, and the age of second language acquisition (Anderson, Mak, Chahi, & Bialystok, 2018; Li, Zhang, Yu, & Zhao, 2019; Luk & Bialystok, 2013). Researchers could then apply multiple linear regression (Plonsky & Oswald, 2017) or Bayesian inference (Ross & Mackey, 2015) to explore whether language skills can predict the age of dementia symptom onset and diagnosis. Researchers can apply objective measures for bilingualism (Clare et al., 2016; Estanga et al., 2017), rather than asking participants or family members to self-report language status (Alladi et al., 2013; Chertkow et al., 2010). Formal assessments of second language proficiency while treating the degree of bilingualism as a continuous variable (DeLuca, Rothman, Bialystok, & Pliatsikas, 2019; Laine & Lehtonen, 2018; Luk & Bialystok, 2013) should be applied. Authors have recently developed questionnaires to quantify participants’ language profiles on a continuum (Anderson et al., 2018) and to measure language proficiency, dominance, as well as immersion (Li et al., 2019). Researchers might want to establish a priori whether they wish to measure bilingualism, the ability to speak two languages (Anderson et al., 2018) or multilingualism, the ability to speak three or more languages (Li et al., 2019). Clearly reporting participants’ immigration status will also be beneficial.

Increasing statistical power will enable partitioning of participants into different dementia etiologies (Nelson et al., 2019) and to conduct sub-group analyses. While categorizing participants into dementia subtypes poses several challenges (De Reuck et al., 2016), applying biomarkers could help researchers in classifying dementia subtypes (Jack et al., 2016; Perneczky et al., 2016). Future studies should consider adjusting for variables such as physical activity, which is associated with the risk of dementia (Brini et al., 2018b). Researchers wishing to adjust for genetic risk would need to recruit a large number of mono- and bilinguals to reach sufficient statistical power for this type of analysis (Sham & Purcell, 2014) and exclude participants for which the *APOE* ε4 may not be clinically relevant (Lovati et al., 2010). Additionally, while bilinguals vs. monolinguals were older at dementia diagnosis, the observed delay does not imply disease-modifying effects (Galimberti & Scarpini, 2016). Combining behavioral measures with surrogate biomarkers such as brain data will provide more robust evidence as to whether bilingualism can help maintain cognitive function despite presence of neuropathology due to dementia (Bialystok, Anderson, & Grundy, 2018; Brini et al., 2018a) and could reveal potentially disease-modifying properties (Galimberti & Scarpini, 2016). Researchers wishing to conduct conditional power calculations for future studies based on our meta-analyses need to take into consideration heterogeneity when estimating sample size (Roloff, Higgins, & Sutton, 2013). Finally, to enhance reporting for observational studies, authors should follow and clearly state that their study adhered to the Strengthening the Reporting of Observational Studies in Epidemiology (STROBE) Statement (Von Elm et al., 2014).

### Implications and Conclusion

Identifying factors that can delay the onset of dementia and AD is a major public health priority (Winblad et al., 2016; Wortmann, 2012). This is because, a delay in the onset of AD of five years could reduce AD prevalence by 57% with concomitant savings of US$627 to US$344 billion in Medicare costs worldwide (Sperling et al., 2011). At the individual level, delaying the symptom onset of dementia and AD can also have important benefits for patients, families, and, by implication, the overall incidence of AD (Cummings, Morstorf, & Zhong, 2014). Our findings suggest that speaking two or more languages may be related to an ability to maintain functional cognition for a longer time compared to monolingualism. The observed effect sizes may be superior, under certain settings, to available pharmacological therapies that delay cognitive decline by 6–12 months and only target symptoms without modifying the pathogenic or clinical course of AD (Yiannopoulou & Papageorgiou, 2013).

While bilingualism appears to be associated with delayed AD symptom onset, dementia and AD diagnosis, the substantial heterogeneity and several sources of bias challenge the interpretation of our findings. Until future studies improve the measurement of participants’ language profiles, increase sample sizes, comprehensively report sample characteristics including participants’ ethnicity and birthplace, adjust for baseline dementia and AD risk (separately), it will be problematic to discern under which settings and to what extent bilingualism may be beneficial. Precisely because of these unanswered questions, it is premature to take a stance on the relevance of bilingualism as a way to delay dementia. We also disagree that longitudinal prospective studies were “large high quality prospective studies” (Mukadam et al., 2017). We argue that longitudinal prospective studies were likely underpowered and carried serious methodological limitations and that, it is incorrect to conclude *evidence of no effect* (Mukadam et al., 2017) from *no evidence of an effect* (Schünemann et al., 2019). Given that the observed effect sizes may be superior to available pharmacological therapies (Yiannopoulou & Papageorgiou, 2013), we agree with others that researchers should improve study methodology and continue investigating the link between bilingualism and dementia (Del Maschio et al., 2018).
