# Correction to: Bilingualism Is Associated with a Delayed Onset of Dementia but Not with a Lower Risk of Developing it: a Systematic Review with Meta-Analyses

**DOI:** 10.1007/s11065-020-09435-7

**Published:** 2020-03-13

**Authors:** Stefano Brini, Hamid R. Sohrabi, Jeffrey J. Hebert, Mitchell R. L. Forrest, Matti Laine, Heikki Hämäläinen, Mira Karrasch, Jeremiah J. Peiffer, Ralph N. Martins, Timothy J. Fairchild

**Affiliations:** 1 Discipline of Psychology, Exercise Science, Chiropractic and Counselling, Murdoch University, Perth, Western Australia Australia; 2 Turku Brain and Mind Center, Turku, Finland; 3 Health Services Research and Management School of Health Sciences, City, University of London, London, UK; 4 School of Medical and Health Sciences, Edith Cowan University, Perth, Western Australia Australia; 5 Department of Biomedical Sciences, Macquarie University, Macquarie Park, New South Wales Australia; 6 Faculty of Kinesiology, University of New Brunswick, Fredericton, Canada; 7 Department of Psychology, Åbo Akademi University, Turku, Finland; 8 Australian Alzheimer’s Research Foundation, Perth, Western Australia Australia; 9 Centre for Molecular Medicine and Innovative Therapeutics, Murdoch University, Perth, Western Australia Australia; 10 Department of Psychology and Speech-Language Pathology, University of Turku, Turku, Finland

**Correction to: Neuropsychology Review**

10.1007/s11065-020-09426-8

The original version of this article unfortunately contained the following mistakes.

1. In the **Results** section under the paragraph **Disease Severity**, the sentence “The PIs ranged between -0.47 and 0.57 MMSE points” should read −0.49 and 0.59 MMSE points.
2. In Figs. 3, 5, and 7, the labels “favour bilinguals” and “favours monolinguals” should be inverted. Therefore, it should be “favours monolinguals” and “favours bilinguals”. Please see below for the correct figures.

**Fig. 3:**
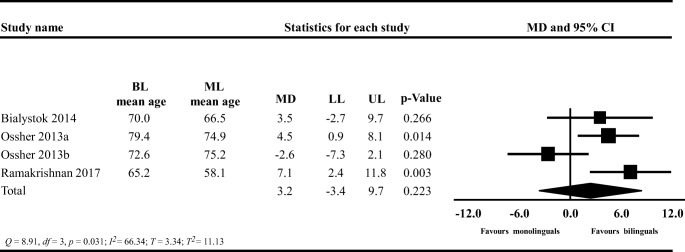
Forest plot showing the mean difference (MD) in the age of MCI diagnosis between bilinguals (BL) and monolinguals (ML); MCI: Mild cognitive impairment; LL: lower limit, UP: upper limit; CI: confidence interval

**Fig. 5:**
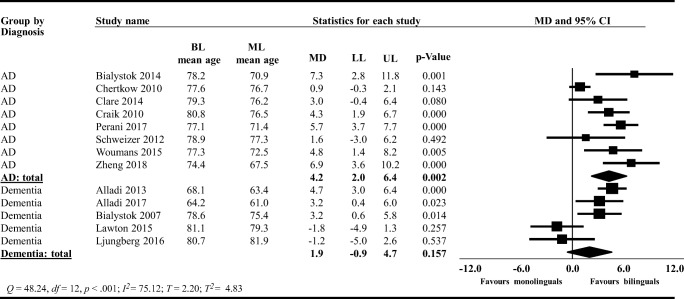
Forest plot showing the mean difference (MD) in the subgroup meta-analysis comparing studies including participants with AD to studies including participants with dementia on the age of AD and dementia diagnosis between bilinguals (BL) and monolinguals (ML); AD: Alzheimer’s disease; LL: lower limit, UP: upper limit; CI: confidence interval

**Fig. 7:**
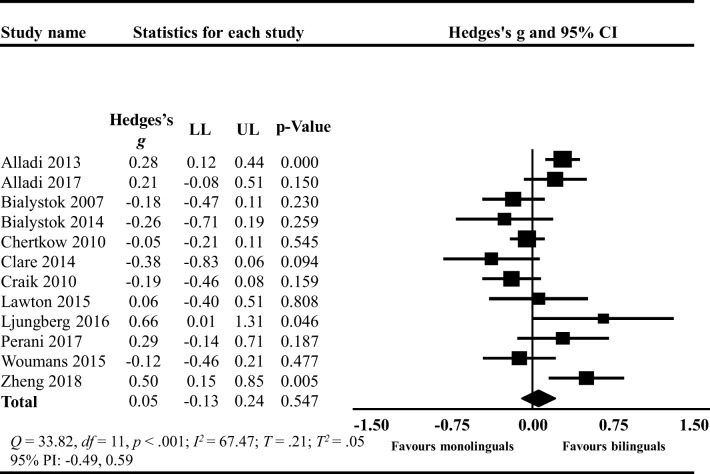
Forest plot showing the standardized mean difference (Hedges’s *g*) in the degree of disease severity at dementia diagnosis between bilinguals (BL) and monolinguals (ML); LL: lower limit, UP: upper limit; CI: confidence interval

